# Predicting local malaria exposure using a Lasso-based two-level cross validation algorithm

**DOI:** 10.1371/journal.pone.0187234

**Published:** 2017-10-31

**Authors:** Bienvenue Kouwaye, Fabrice Rossi, Noël Fonton, André Garcia, Simplice Dossou-Gbété, Mahouton Norbert Hounkonnou, Gilles Cottrell

**Affiliations:** 1 Université Paris 1 Panthéon Sorbonne, Laboratoire SAMM, EA 4543, Paris, France; 2 Université d’Abomey-Calavi, International Chair in Mathmatical Physics and Applications (ICMPA - UNESCO-Chair), 072 BP 50 Cotonou, Republic of Benin; 3 Université d’Abomey-Calavi, Laboratoire d’étude et de recherche en statistique appliquée et biométrie (LERSAB), Republic of Benin; 4 Institut de Recherche pour le Développement, UMR216 MERIT, Mère et enfant face aux infections tropicales, Paris, 75006, France; 5 Faculté de Pharmacie, Université Paris Descartes, Sorbonne Paris Cité, Paris, 75270, France; 6 IRD, UMR 216, Centre d’Etude et de Recherche sur le Paludisme Associé À la Grossesse et À l’Enfance (CERPAGE); Faculté des Sciences de la Santé, Cotonou, Bénin; 7 Université de Pau et des Pays de l’Adour / CNRS, Laboratoire de Mathématiques et de leurs Applications de Pau - Fédération IPRA, UMR 5142, 64012 Pau, France; Colorado State University, UNITED STATES

## Abstract

Recent studies have highlighted the importance of local environmental factors to determine the fine-scale heterogeneity of malaria transmission and exposure to the vector. In this work, we compare a classical GLM model with backward selection with different versions of an automatic LASSO-based algorithm with 2-level cross-validation aiming to build a predictive model of the space and time dependent individual exposure to the malaria vector, using entomological and environmental data from a cohort study in Benin. Although the GLM can outperform the LASSO model with appropriate engineering, the best model in terms of predictive power was found to be the LASSO-based model. Our approach can be adapted to different topics and may therefore be helpful to address prediction issues in other health sciences domains.

## Introduction

Malaria is endemic and remains a major cause of mortality especially for children under the age of five years in sub-Saharan Africa [1]. Assessment of malaria burden is critical for the evaluation of control measures. The correct definition of “unexposed” individuals (not in contact with the malaria vector and then also not with the parasite) is important for the interpretation of results, since it may help in distinguishing protection (i.e immune individuals) from lack of exposure [2]. A precise characterization of exposure could mitigate classification error and facilitate clinical trial and cohort study designs. The exposure to the malaria vector (the Anopheles mosquito) is space and time dependent in endemic areas and highly related to the rainy Season. Recent studies have highlighted the importance of local environmental factors to determine the local-scale heterogeneity of malaria transmission and exposure to the vector bite [3–6]. A classical entomological indicator used to characterize the human exposure to the malaria vector is the human biting rate (hbr), which is the number of anopheles bites per man per time unit. In a previous work, we have built a predictive model to estimate the individual hbr in a population of Beninese children by using entomological and environmental data from a cohort study carried out between 2007 and 2010. Variable selection in statistical models is a highly complex and vast research area with a huge literature [7–15]. In health sciences, regression models are commonly used, but classical variable selection methods (backward, forward selection…) show limits (non convergence, collinearity…) as the ratio variables/observations increases. In particular, taking into account all the interactions terms in GLM with backward selection is often impossible in practice, although it can be useful to improve the prediction power. In our previous work, the selection of the variables introduced in the General Linear Model regression (GLM) was perfomed with a backward procedure and only few interactions terms could have been entered in the model based on an empirical expertise [3]. Machine learning is a growing field of research, particularly adapted for prediction problems in high dimension, and constitutes then an appealing approach to overcome this issue. Several recent studies in biology, epidemiology and medicine have actually shown that the predictive performance of classical methods can be improved by implementing machine learning methods, for example [16], [17–19], [20–22].

The present work aims to revisit the empirical algorithm and to propose an automatic machine learning method combining GLM-Lasso and a stratified two-levels cross validation in order to select the best subset of predictors. The Lasso method proposed by Tibshirani [7] is a regularized estimation approach for regression model using an L1-norm and constraining the regression coefficients, which simultaneously performs selection and estimation, and is robust for variables selection in high dimension [8, 23]. The algorithms implemented in our work are based on [8, 23, 24]. The predictive performances of the automatic LASSO-based method and the reference method are evaluated and compared to each other.

## Materials and methods

### Materials

In this section, we briefly recall the description of the study area, the mosquito collection and related variables. For more details, see [3].

#### Study area

The study was conducted in the district of Tori-Bossito (Republic of Benin), from July 2007 to July 2009. Tori-Bossito is on the coastal plain of Southern Benin, 40 kilometers north-east of Cotonou. This area has a subtropical climate and during the study, the rainy Season lasted from May to October. Average monthly temperatures varied between 27°C and 31°C. The original equatorial forest has been cleared and the vegetation is characterized by bushes with sparse trees, a few oil palm plantations, and farms. The study area contained nine villages (Avamé centre, Gbédjougo, Houngo, Anavié, Dohinoko, Gbétaga, Tori Cada Centre, Zébè, and Zoungoudo). Tori Bossito was recently classified as mesoendemic with a clinical malaria incidence of about 1.5 episodes per child per year [25]. Pyrethroid-resistant malaria vectors are present [26].

#### Mosquito collection and identfication

Entomological surveys based on human landing catches (HLC) were performed in the nine villages every six weeks for two years (July 2007 to July 2009). Mosquitoes were collected at four catch houses in each village over three successive nights (four indoors and four outdoors, i.e. a total of 216 nights every six weeks in the nine villages). Five catch sites had to be changed in the course of the study (2 in Gbedjougo, 1 in Avamè, 1 in Cada, 1 in Dohinoko) and a total of 19 data collections was performed in the field from July 2007 to July 2009. In total, data from 41 catch sites are available. Each collector caught of predictional mosquitoes landing on the lower legs and feet between 10 pm and 6 am. All mosquitoes were held in bags labeled with the time of collection. The following morning, mosquitoes were identified on the basis of morphological criteria [27, 28]. All *Anopheles gambiae* complex and *Anopheles funestus* mosquitoes were stored in individual tube with silica gel and preserved at 220°C. *Plasmodium falciparum* infection rates were then determined on the head and thorax of individual anopheline specimens by CSP-ELISA [29].

#### Environnement and behavioral data

Original variables: Rainfall was recorded twice a day with a pluviometer in each village. In and around each catch site, the following information was systematically collected: (1) type of soil (dry lateritic or humid hydromorphic)-assessed using a soil map of the area (map IGN Benin at 1/200 000 e, sheets NB-31-XIV and NB-31-XV, 1968) that was georeferenced and input into a GIS; (2) presence of areas where building constructions are ongoing with tools or holes representing potential breeding habitats for anopheles; (3) presence of abandoned objects (or ustensils) susceptible to be used as oviposition sites for female mosquitoes; (4) a watercourse nearby; (5) number of windows and doors; (6) type of roof (straw or metal); (7) number of inhabitants; (8) ownership of a bed-net or (9) insect repellent; and (10) normalized difference vegetation index (NDVI) which was estimated for 100 meters around the catch site with a SPOT 5 High Resolution (10 m colors) satellite image (Image Spot5, CNES, 2003, distribution SpotImage S.A) with assessment of the chlorophyll density of each pixel of the image S1 Database. Due to logistical problems, rainfall measurements are only available after the second entomological survey. Consequently, we excluded the first and second survey (performed in July and August 2007 respectively) from the statistical analyses.

Recoded variables: Some pre-treatments based on the knowledge of experts in entomology and medicine are operated on some original variables. These pre-treatments generate a second type of covariables called recoded variables. The dependent variable was the number of Anopheles collected in a house over the three nights of each catch and the explanatory variables were the environmental factors, i.e. the mean rainfall between two catches (classified according to quartile), the number of rainy days in the ten days before the catch (3 classes [0–1], [2–4], >4 days), the Season during which the catch was carried out (4 classes: end of the dry Season from February to April; beginning of the rainy Season from May to July; end of the rainy Season from August to October; beginning of the dry Season from November to January), the type of soil 100 meters around the house (dry or humid), the presence of constructions within 100 meters of the house (yes/no), the presence of abandoned tools within 100 meters of the house (yes/no), the presence of a watercourse within 500 meters of the house (yes/no), NDVI 100 meters around the house (classified according to quartile), the type of roof (straw or Sheet metal), the number of windows (classified according to quartile), the ownership of bed nets (yes/no), the use of insect repellent (yes/no), and the number of inhabitants in the house (classified according to quartile).

The Original and the recoded variables are described in Tables 1 and 2. Two groups of covariables set are used: the first group (Group 1), the original covariables with all covariables obtained by interactions, the second group (Group 2), the recoded covariables with all covariables obtained by interactions.

**Table 1:** Description of original variables.

Variable	Nature	Number of modalities	Modalities
Repellent	Non-numeric	2	Yes/ No
Bed-net	Non-numeric	2	Yes/ No
Type of roof	Non-numeric	2	Sheet metal/ Straw
Ustensils	Non-numeric	2	Yes/ No
Presence of constructions	Non-numeric	2	Yes/ No
Type of soil	Non-numeric	2	Humid/ Dry
Water course	Non-numeric	2	Yes/ No
Season	Non-numeric	4	1/2/3/4
Village	Non-numeric	9	
House	Non-numeric	41	
Rainy days before mission	Numeric	Discrete	0/2/⋯/9
Rainy days during mission	Numeric	Discrete	0/1/⋯/3
Fragmentation Index	Numeric	Discrete	26/⋯/71
Openings	Numeric	Discrete	1/⋯/5
Number of inhabitants	Numeric	Discrete	1/⋯/8
Mean rainfall	Numeric	Quantitative	0/⋯/82
Vegetation	Numeric	Quantitative	115.2/⋯/ 159.5
Total Mosquitoes	Numeric	Discrete	0/⋯/481
Total Anopheles	Numeric	Discrete	0/⋯/87
Anopheles infected	Numeric	Discrete	0/⋯/9

Season: 1, beginning of dry Season; 2, end of rainy Season; 3 beginning of rainy Season; 4, end of dry Season.

**Table 2:** Description of recoded variables. Variables with star are recoded.

Variable	Nature	Number of modalities	Modalities
Repellent	Non-numeric	2	Yes/ No
Bed-net	Non-numeric	2	Yes/ No
Type of roof	Non-numeric	2	Sheet metal/ Straw
Utensils	Non-numeric	2	Yes/ No
Presence of constructions	Non-numeric	2	Yes/ No
Type of soil	Non-numeric	2	Humid/ Dry
Water course	Non-numeric	2	Yes/ No
Season	Non-numeric	4	1/2/3/4
Village*	Non-numeric	9	
House*	Non-numeric	41	
Rainy days before mission*	Non-numeric	3	Quartile
Rainy days during mission	Numeric	Discrete	0/1/⋯/3
Fragmentation index*	Non-numeric	4	Quartile
Openings*	Non-numeric	4	Quartile
Nber of inhabitants*	Non-numeric	3	Quartile
Mean rainfall*	Non-numeric	4	Quartile
Vegetation*	Non-numeric	4	Quartile
Total Mosquitoes	Numeric	Discrete	0/⋯/481
Total Anopheles	Numeric	Discrete	0/⋯/87
Anopheles infected	Numeric	Discrete	0/⋯/9

Season: 1, beginning of dry Season; 2, end of rainy Season; 3, beginning of rainy Season; 4, end of dry Season.

### Methods

#### Ethics

A written informed consent was obtained from all participants involved in the study. The study protocol was approved by the Ethics Committee of the University of Abomey-Calavi (Faculté des Sciences de la Santé; FSS) in Benin and the Consultative Committee of Ethics of Institute of Development Research (IRD).

#### Methods

The main objective is to predict the number of Anopheles *Y* using the environmental factors *X*.

$$\begin{array}{c} \ln[E(Y|X,\beta )]=X\beta \end{array}$$ (1)

For doing this, statistical analysis are conducted in three steps.

#### Step 1

First, the variables selection is performed using GLM-lasso method through a cross validation. For this part, we have implemented an automatic algorithm Leave One Level Out Double Cross-Validation (LOLO-DCV) 0.1. This algorithm developed in this work is a stratified cross validation with two levels [30, 31].

**Algorithm 0.1**

1. The data are separated in *N*_*f*_-folds

2. At each first level *k*

- The folds are regrouped in two parts: *A*_*k*_ and *E*_*k*_, *A*_*k*_: the learning set containing the observations of (*N*_*f*_ − 1)-folds, *E*_*k*_: the test set, containing the observations of the last fold.
- Holding-out *E*_*k*_
- The second level of cross validation
  - A full cross validation is computed on *A*_*k*_
  - The two regularizing parameters *λ*.*min*_*k*_ and *λ*.1*se*_*k*_ are obtained.
  - The coefficients of active variables i.e variables with non-zero coefficients associated to these two parameters are debiased
  - Predictions are performed using a GLM model on *E*_*k*_
  - The presence $P(X_{i})$ of each variable is determined using *λ*.*min*_*k*_ and *λ*.1*se*_*k*_ on *A*_*k*_

3. The step 2c is repeated until predictions are performed for all observations.

The second level allows to avoid over-fitting in learning stage in the process of variables selection because the number of observations is lower. Its aim is to compute a second cross validation (*CV*_2_) for prediction at each step of learning of a first cross validation (*CV*_1_). The GLMM-Lasso method of variables selection is based on the calculation of the coefficients of the variables defined as:

$$\begin{array}{c} \hat{\beta }\left(\lambda \right)=Arg\max_{\beta }\left[L_{GLM}\left(\beta |\;D\right)-\lambda \sum_{j}|\beta_{j}|\right] \end{array}$$ (2)

$D=\{\left(Y=y_{i},X=x_{i}\right),1\leq i\leq n\}$, *X* is the *n*×(*p*+1)-dimension matrix of covariables (environmental variables), *n* is the number of observations, *p* is the number of covariables, *β* is a (*p*+1)-vector of fixed parameters including the intercept, *Y* is the vector of the target variable, *L*_*GLM*_ the likelihood of the model, *λ* is the regularizing parameter, The choice of the regularizing parameter lambda is done by minimizing a score function based on the deviance. In practice, Eq (2) is solved using a combination of Laplace approximation, Newton-Raphson method or Fisher scoring method. The deviance can be defined as:

$$\begin{array}{c} Deviance(M(\beta ))=2(L(M\left(sat\right))-L(M\left(\beta \right))) \end{array}$$ (3)

where L(M(β)) the log-likelihood of the model M(β), M(sat) is the “saturated” model and M(β) is the model of Poisson regression. The selection of the best subset of variables is done according to two strategies, LDLM (Lolo Dcv Lambda Min) and LDLS (Lolo Dcv Lambda 1Se). The strategy LDLM is based on the regularizing parameter defined as:

$$\begin{array}{c} \lambda .min=Arg\min_{\lambda_{k}}[Deviance\left(M\left(\left(\hat{\beta }\left(\lambda_{k}\right)\right)\right)\right]. \end{array}$$ (4)

The strategy LDLS is based on the value *λ*.1*se* defined by T. Hastie et *al* which minimizes the deviance plus its standard deviation [23, 32, 33]:

$$\begin{array}{c} \lambda .1se=Arg\min_{\lambda_{k}}\left[Deviance\left(M\left(\hat{\beta }\left(\lambda_{k}\right)\right)\right)+Std\left(Deviance\left(M\left(\hat{\beta }\left(\lambda_{k}\right)\right)\right)\right)\right]. \end{array}$$ (5)

The best subset of variables is selected as follows. Let $V=\{V_{1},V_{2},\ldots ,V_{N_{var}}\}$ be the set of all variables including interactions, *N*_*var*_ the number of variables. If *N*_*f*_ is the number of folds, at each first level *k*, 1 ≤ *k* ≤ *N*_*f*_, the second level of cross validation provides two vectors *β*(*λ*.*min*_*k*_) and *β*(*λ*.1*se*_*k*_) of coefficients of variables using *λ*.*min*_*k*_ and *λ*.1*se*_*k*_ Eqs (4) and (5) respectively. Based on this, one can determine the presence or the absence of each covariable. For any *λ*, let define the function “Presence” of variable like:

$$\begin{array}{c} \left\{ \begin{array}{cc} P_{k}\left(V_{r}\right)=1 & if\;\;\;\beta_{r}\left(\lambda \right)\neq \Theta \;\\ P_{k}\left(V_{r}\right)=0 & \text{elsewhere} \end{array}\right. \end{array}$$

where *β*_*r*_(*λ*), 1 ≤ *r* ≤ *N*_*var*_ is a vector of coefficients of covariables *V*_*r*_ and Θ the null vector. For a threshold *s*, 1 ≤ *s* ≤ 100, the subset of selected variables (SV) is defined as:

$$\begin{array}{c} \text{SV}\left(\lambda ,s\right)=\left\{V_{r},\frac{100}{N_{f}}\times \sum_{k=1}^{N_{f}}P_{k}\left(V_{r}\right)\geq s\right\}. \end{array}$$ (6)

We also studied the influence of the variability of the threshold *s* on the quality of the prediction. Then we compared the predictive performance of the model for *s* taken in {75, 80, 90, 95, 100}. At the end of this step the strategies LDLM and LDLS provides two optimal subset of variables SV_*LDLM*_, and SV_*LDLS*_ which are used to build a GLM predictive model.

#### Step 2

The predictive performance of the models described above are compared to each other and to the reference B-GLM model The comparison criteria are:

1. The mean of predictions
2. The quadratic risk of predictions
3. The absolute risk of predictions

## Results

### Summary of results on prediction accuracy and quality criteria with LOLO-DCV

The Tables 3 and 4 present the comparison of the performance of the three models B-GLM, LDLM, and LDLS models in terms of quadratic and absolute risks. When selection and prediction are performed using the recoded variables, the reference B-GLM model is the best regarding the indicators of performance for any threshold. On the other hand, when selection and prediction are performed using the original variables, LDLM and LDLS are superior to B-GLM but only with a 100% threshold.

**Table 3:** Summary of predictions for B-GLM, LDLM, and LDLS on original variables.

Threshold	Strategy	Mean	Quadratic risk	Absolute risk
-	B-GLM	3.75	62.20	3.81
100	**LDLM**	**3.74**	**44.26**	**3.30**
	LDLS	3.74	54.50	3.62
95	LDLM	3.74	72.01	4.42
	LDLS	3.74	72.03	4.40
90	LDLM	3.74	72.00	4.47
	LDLS	3.75	72.01	4.42
80	LDLM	3.75	74.00	4.71
	LDLS	3.72	73.02	4.52
75	LDLM	3.74	71.84	4.41
	LDLS	3.74	72.00	4.31

**Table 4:** Summary of predictions for B-GLM, LDLM, and LDLS on recoded variables.

Threshold	Strategy	Mean	Quadratic risk	Absolute risk
	B-GLM	3.75	62.29	3.88
100	LDLM	3.85	82.06	4.67
	LDLS	3.76	74.08	4.76
95	LDLM	3.84	81.06	4.61
	LDLS	3.76	74.08	4.76
90	LDLM	3.87	83.06	4.72
	LDLS	3.75	75.07	4.86
80	LDLM	3.87	84.06	4.81
	LDLS	3.75	75.07	4.86
75	LDLM	3.89	84.05	4.79
	LDLS	3.77	75.56	4.85

### Optimal subset of variables for prediction

Tables 5, 6, and 7 show that both of the strategies LDLM and LDLS provide a sparse optimal subset for original variables.

**Table 5:** Frequency of original stable covariables.

Variable	Frequency for LDLM (%)	Frequency for LDLS (%)
Season	100	100
Mean rainfall: Openings	100	80
Rainy days before mission: Nbr of inhabitants	100	-
Rainy days during mission: Vegatation	100	95
Season: water course	95	-
Season: Type of Soil	95	-
Season: Village	95	-
Mean rainfall: Vegetation	95	-
Rainy days durin mission: Village	90	-
Season:Rainy days durin mission	80	-
Season: Repellent	75	-
Season: Presence of construction	75	-

**Table 6:** Number of original stable covariables for the strategies LDLM and LDLS.

Threshold (%)	Number for LDLM	Number for LDLS
100	4	1
95	8	2
90	9	2
80	10	3
75	12	3

**Table 7:** Number of recoded stable covariables for the strategies LDLM and LDLS.

Threshold (%)	Number for LDLM	Number for LDLS
100	31	11
95	39	11
90	44	16
80	50	22
75	52	29

**Fig 1:**
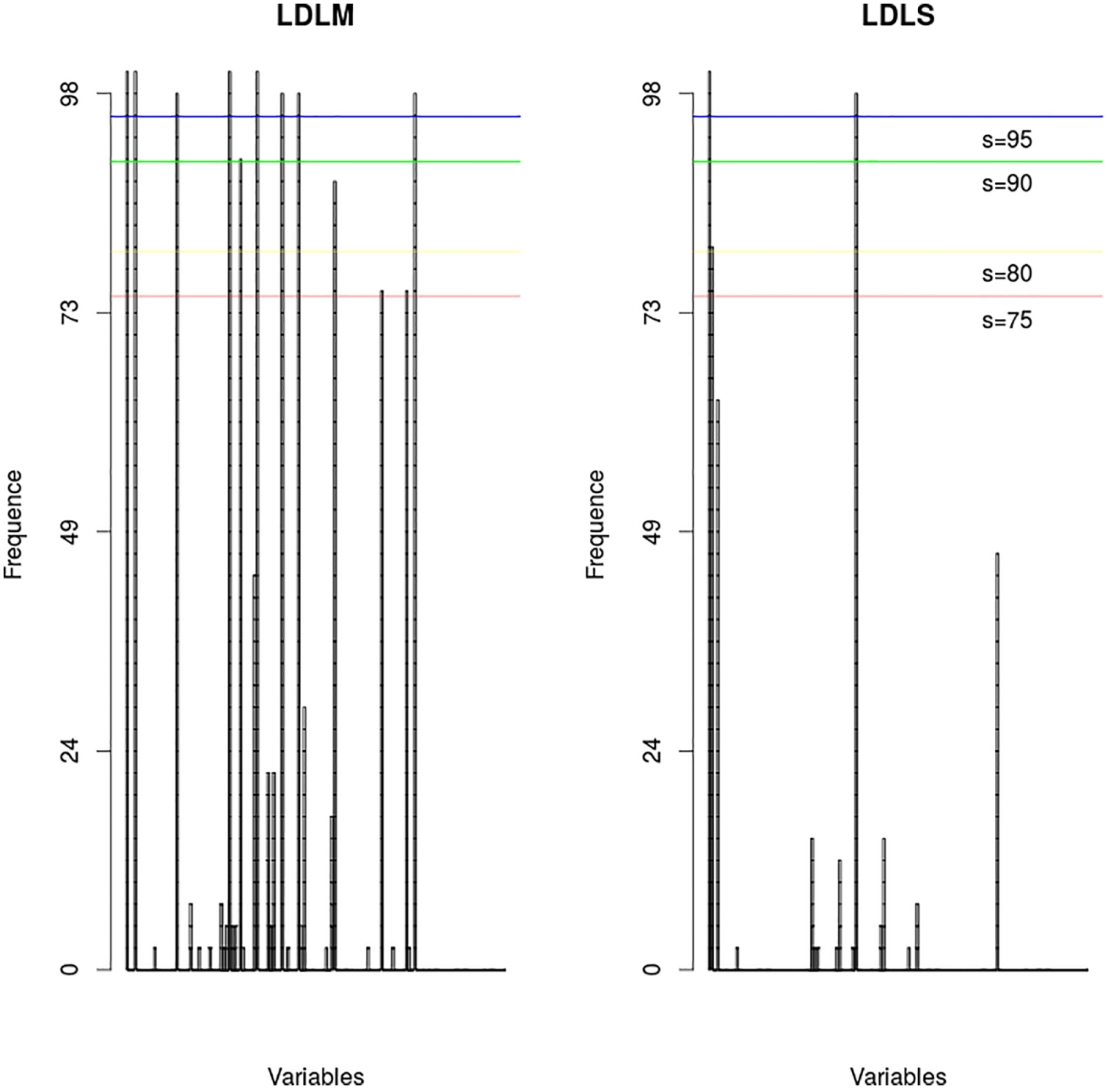
Frequent variables. The x-axis shows the variables including the interactions, and the y-axis shows the percentage of presence of the variables. The left figure corresponds to the LDLM strategy and the right figure corresponds to LDLS strategy. Each vertical band represents one variable.

The best subset of variables selected for each group of covariable is:

1. **B-GLM**
  For B-GLM [3], the best subset of covariables is Season, Number of rainy during the mission, Mean rainfall, Rainy days before mission, Repellent, Vegetation, the interaction between Season and Vegetation.
2. **LOLO-DCV (LDLM and LDLS)**
  Based on the results of Fig 1 and the Tables 3, 4, and 5, the best covariables for the Group 1 and Group 2 is: Season; interaction between Mean rainfall and openings; interaction between Rainy days before mission and Nbr of inhabitants; interaction between Rainy days during the mission and Vegetation

**Fig 2:**
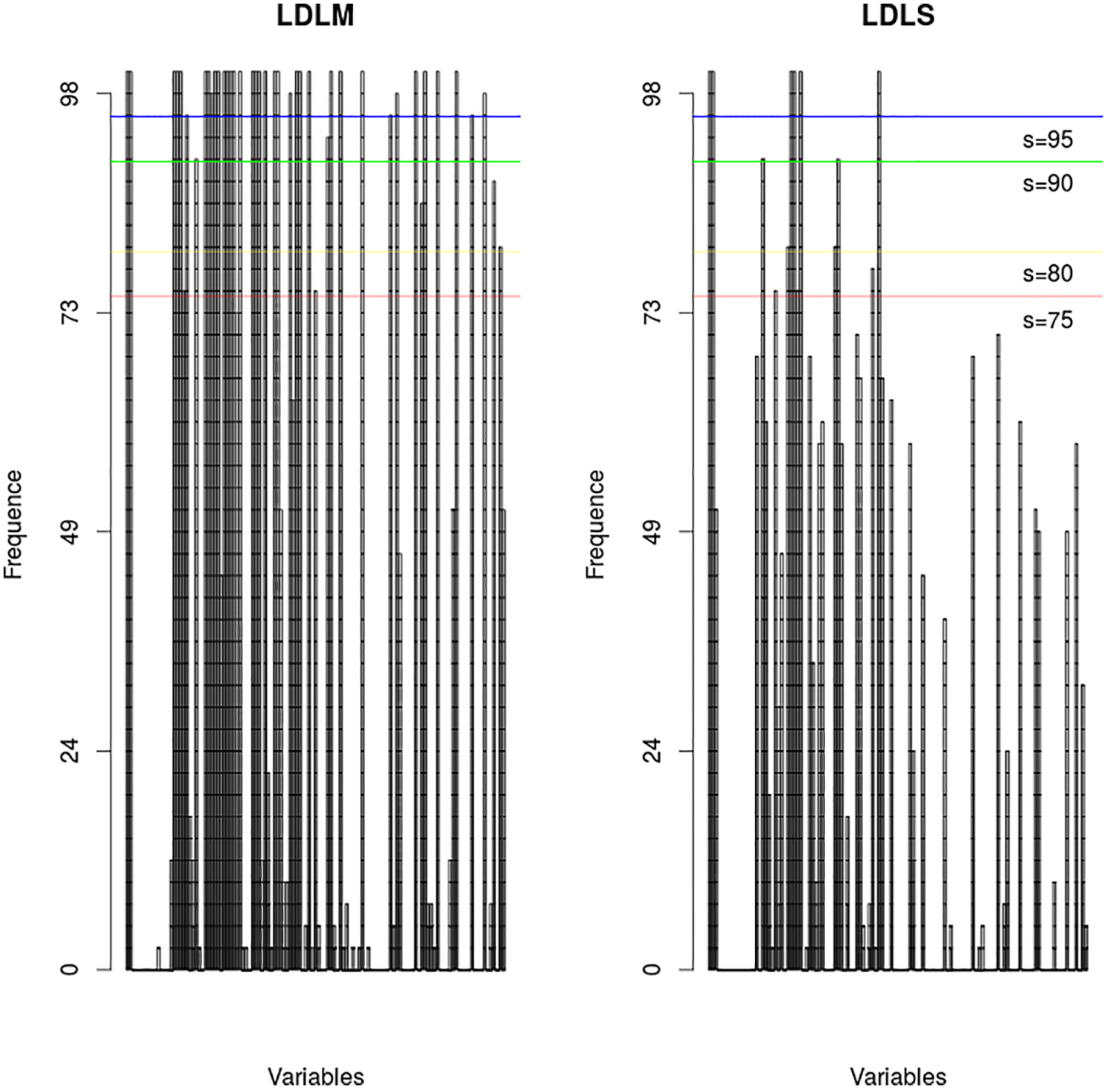
Frequent variables. The x-axis shows the variables including the interactions, and the y-axis shows the percentage of presence of the variables. The left figure corresponds to the LDLM strategy and the right figure corresponds to LDLS strategy. Each vertical band represents one variable.

**Fig 3:**
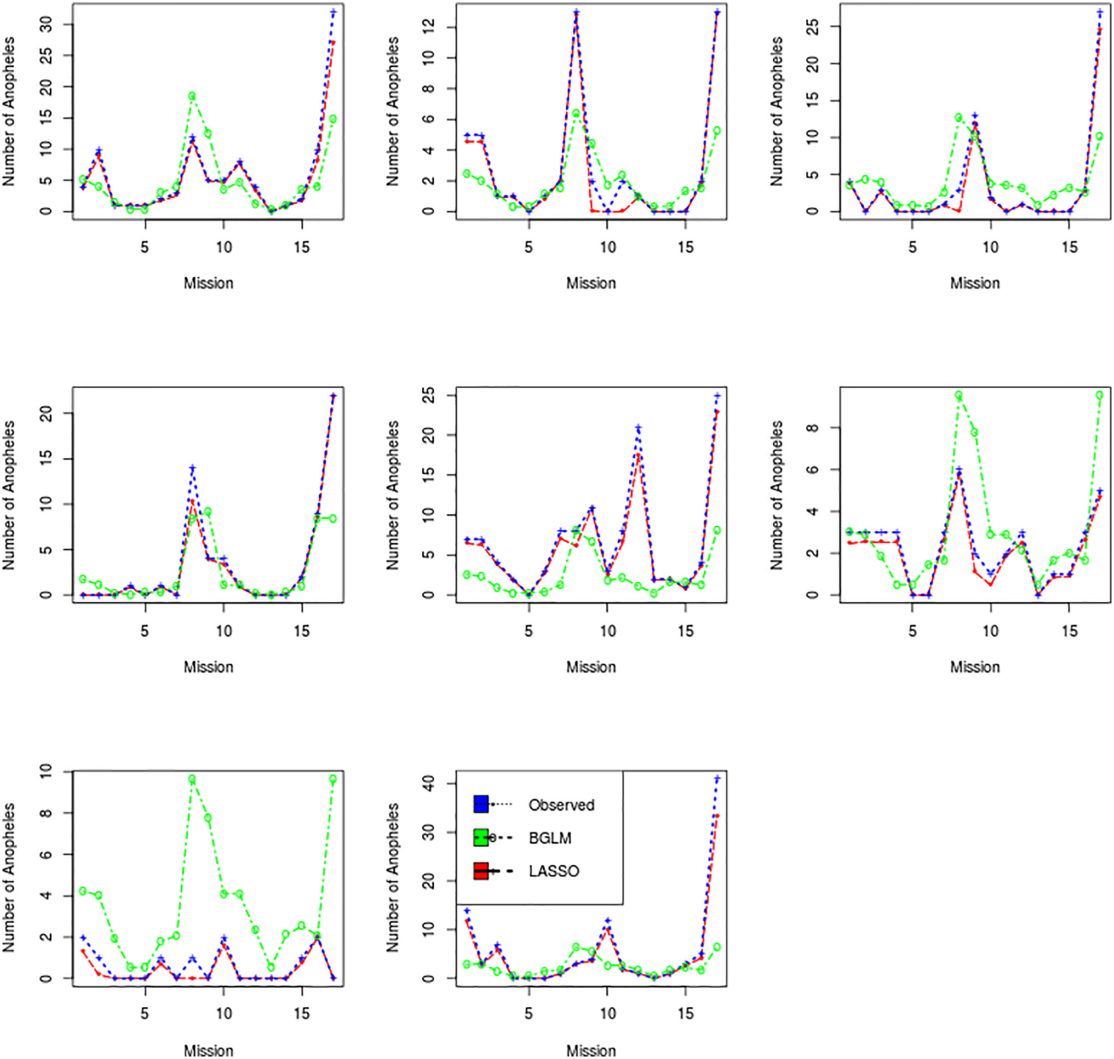
Comparison between observed and predicted number of anopheles in eight houses. The line with “⋆” is for observed values, the line with “o” is for B-GLM and the line with “+” is for LOLO-DCV.

Figs 1 and 2, show how the number of selected variables is reduced as the threshold increases. The fact that the best model is obtained for a threshold equal to 100% (LDLM strategy) shows that the best prediction power is reached when unstable variables are removed from the final subset. Fig 3, show the number of mosquitoes collected at every mosquito collection mission at 4 collection sites (given as example of the 41 collection sites) predicted by the B-GLM model, predicted by the LDLS model, and the observations (the number of mosquitoes actually caught). The LDLS model predictions were better than the B-GLM model ones, and the predictive curve from LDLS is often able to mimic the observations curves in a very satisfactory way. This has been found for the highest majority of the 41 collection sites.

## Discussion and conclusion

The main objective of this work was to propose an automatic algorithm (LOLO-DCV) based on a machine learning approach for variables selection and prediction of the malaria exposure from data of a cohort study carried out in Benin. This algorithm has been performed using both the original variables and then the variables recoded based on the expert knowledge of the topic, and its prediction power has been compared to an empirical algorithm previously used on the same data [3]. This automatic algorithm has shown a substantial improvement in terms of predictive power compared to the empirical algorithm.

Our LOLO-DCV algorithm has several advantages on the reference empirical variable selection method (B-GLM). First, being based on the LASSO method, the high ratio variables/observations is no longer an issue and all the variables can be entered together in the model, including all their second order interactions (automatically generated). This avoids the subjective part of the empirical analysis where a pre-selection based on the field expertise is needed to limit the variables/observations ratio. Second, the algorithm is automatically performed in a reasonable CPU-time (on our data), while the empirical algorithm would require much more time manually. Third, the second level of cross-validation makes this method more robust (and then more generalizable) than the empirical algorithm. Fourth, and most important, LOLO-DCV succeeded to improve the prediction performance of the empirical model, which is of course the ultimate goal. We observe that the global performance criteria as well as the local predictive power at the house (collection site) level are substantially improved compared to the empirical algorithm. In particular, LOLO-DCV algorithm was able to improve two important drawbacks that were observed for prediction at the house level by the reference method: (i) the extreme values were hardly reached by the B-GLM predictions and are much better predicted by LOLO-DCV and (ii) LOLO-DCV succeeded for most of the houses to mimic the exact shape of the observations curves, whereas the B-GLM only succeeded to approximate this shape. Overall, all these improvement make LOLO-DCV algorithm a superior alternative to the B-GLM method. Many other machine learning methods exist, for example random forest, boosting regression etc, [34][35–38]. But a drawback of these alternative methods is that they do not lead to easily interpretable results [16, 37, 38], [39]. The interpretation of the results given by the LOLO-DCV method is the same as those from a classical regression model, thereby much easier to understand by the malaria experts than the results from other methods. In particular, the subset of variables and interactions selected by LOLO-DCV is consistent. As expected the rainfall and Season variables are of highest importance, which is relevant.

However, we cannot ensure that our LOLO-DCV algorithm guaranties the best predictive performance, and maybe other approaches would even give better results. This is a limitation of our work and other experiments may be condutcted to explore this matter.

In our work, original variables have shown better results than recoded variables. It may be due to the fact that in our case, recoding was to categorize quantitative variables, which allows to interpret the results more easily, but is known to reduce the variability (and then the information) of the variables. However, it may not be a general result, and we do not recommend avoiding systematically recoded variables.

In conclusion, this work has confirmed the value of using a machine learning approach to address the important health science problem of predicting the individual malaria exposure in a cohort study. Such approach can be helpful to improve the predictive performance of the classical methods and to overcome their limits. Our Lasso-based LOLO-DCV algorithm has clearly shown a substantial improvement compared to the reference method, giving robust and easy-to-interpret results by non-statisticians or machine learning specialists. We think LOLO-DCV can then be recommended to predict any count outcome from a dataset of several dozen of variables and hundreds of observations, which is an average dataset dimension in this study area. For all these reasons the authors plan to build an easy-to-use R package and recommend the use of LOLO-DCV in prediction problem in health science.

## Supporting information

S1 Database S1_Database.xls. This database contains all original environnement and behavioral variables used in this work.
(XLS)
